# Intra- vs. Interhost Evolution of SARS-CoV-2 Driven by Uncorrelated Selection—The Evolution Thwarted

**DOI:** 10.1093/molbev/msad204

**Published:** 2023-09-14

**Authors:** Mei Hou, Jingrong Shi, Zanke Gong, Haijun Wen, Yun Lan, Xizi Deng, Qinghong Fan, Jiaojiao Li, Mengling Jiang, Xiaoping Tang, Chung-I Wu, Feng Li, Yongsen Ruan

**Affiliations:** State Key Laboratory of Biocontrol, School of Life Sciences, Sun Yat-sen University, Guangzhou, China; Guangzhou Eighth People's Hospital, Guangzhou Medical University, Guangzhou, China; State Key Laboratory of Biocontrol, School of Life Sciences, Sun Yat-sen University, Guangzhou, China; State Key Laboratory of Biocontrol, School of Life Sciences, Sun Yat-sen University, Guangzhou, China; Guangzhou Eighth People's Hospital, Guangzhou Medical University, Guangzhou, China; Guangzhou Eighth People's Hospital, Guangzhou Medical University, Guangzhou, China; Guangzhou Eighth People's Hospital, Guangzhou Medical University, Guangzhou, China; Guangzhou Eighth People's Hospital, Guangzhou Medical University, Guangzhou, China; Guangzhou Eighth People's Hospital, Guangzhou Medical University, Guangzhou, China; Guangzhou Eighth People's Hospital, Guangzhou Medical University, Guangzhou, China; State Key Laboratory of Biocontrol, School of Life Sciences, Sun Yat-sen University, Guangzhou, China; Guangzhou Eighth People's Hospital, Guangzhou Medical University, Guangzhou, China; State Key Laboratory of Biocontrol, School of Life Sciences, Sun Yat-sen University, Guangzhou, China

**Keywords:** SARS-CoV-2, intrahost evolution, interhost evolution, antagonism, variants of concern

## Abstract

In viral evolution, a new mutation has to proliferate within the host (Stage I) in order to be transmitted and then compete in the host population (Stage II). We now analyze the intrahost single nucleotide variants (iSNVs) in a set of 79 SARS-CoV-2 infected patients with most transmissions tracked. Here, every mutation has two measures: 1) iSNV frequency within each individual host in Stage I; 2) occurrence among individuals ranging from 1 (private), 2–78 (public), to 79 (global) occurrences in Stage II. In Stage I, a small fraction of nonsynonymous iSNVs are sufficiently advantageous to rise to a high frequency, often 100%. However, such iSNVs usually fail to become public mutations. Thus, the selective forces in the two stages of evolution are uncorrelated and, possibly, antagonistic. For that reason, successful mutants, including many variants of concern, have to avoid being eliminated in Stage I when they first emerge. As a result, they may not have the transmission advantage to outcompete the dominant strains and, hence, are rare in the host population. Few of them could manage to slowly accumulate advantageous mutations to compete in Stage II. When they do, they would appear suddenly as in each of the six successive waves of SARS-CoV-2 strains. In conclusion, Stage I evolution, the gate-keeper, may contravene the long-term viral evolution and should be heeded in viral studies.

**Significance Statement**

In systems that include viruses, new mutations evolve through two stages—within and then between individuals. While the intrahost stage is crucial, the current practice of presenting one DNA sequence per host skips this stage entirely. In a cohort of 79 COVID-19 patients that have a complete contact record, we could track the evolution of SARS-CoV-2 both within and between hosts and, most importantly, the transition between the two stages. We found that advantageous new mutations emerge regularly within individual hosts but rarely succeed in spreading among hosts. The two stages are thus uncorrelated and even antagonistic. The conflicting demands between stages may constrain the evolutionary potentials of viruses, despite their large population sizes.

## Introduction

Selection for new mutations is the essence of molecular evolution (Li 1997). For virus, this phase of selection must happen within a host first. Hence, a study of viral evolution has to consider the selective advantage, or disadvantage, within individuals. We shall refer to this stage of evolution as Stage I. After the mutations sweep through within the host, they compete with the prevalent strains from other individuals in Stage II evolution.

In Stage I, we need to track intrahost single nucleotide variants (iSNVs), which are the alternative alleles at identical genomic position within an intrahost sample. For a de novo mutation in an individual to become detectable as an iSNV, it must increase from one virion in millions to an appreciable frequency beyond the sequencing error rate. Before an iSNV reaches 50% in frequency, it is essentially invisible in the current practice of presenting only one viral genome per individual. This practice explicitly assumes little intrahost variation and directs the focus to Stage II, bypassing Stage I evolution entirely (Korber et al. 2020; Rambaut, Holmes, et al. 2020; Tang et al. 2020; Zeng et al. 2020; Dellicour et al. 2021; Planas et al. 2021; Ruan, Luo, et al. 2021).

Presenting one genome per host can be justified if the number of virions that successfully colonize a new host (denoted *N*_0_) is very small. Obviously, with *N*_0_ = 1, there is no within-host diversity at the start of infection. Note that *N*_0_ should be much smaller than the number of virions in the droplets or aerosol carrying the virus (Killingley et al. 2022; Puhach et al. 2022). While *N*_0_ has been frequently estimated to be close to 1 (Braun et al. 2021; Lythgoeet al. 2021; Martin and Koelle 2021; Wang, Wang, et al. 2021), others have shown that *N*_0_ is large enough to preserve the intrahost polymorphism during transmission (Popa et al. 2020; Ruan, Hou, et al. 2021). The difference in estimates is mainly due to de novo mutations in the donors (as well as the recipients), which are not involved in the transmission and should be excluded from the estimation of *N*_0_.

While tracking iSNVs is necessary for a full understanding of viral evolution, iSNVs also have clinical values. Viral strains that have spread widely and displayed detrimental effect on human health have been classified as variants of concern (VOCs), including Delta and Omicron (WHO). VOCs are reported only when their characteristic mutations become high-frequency (>50%) iSNVs. However, these mutations may be detectable at lower frequencies within hosts long before VOCs are identified. Despite the unprecedented efforts in surveillance, the lack of intermediate sequences has prevented us from accurately describing how the VOCs emerge (Ruan, Wen, et al. 2021; Wu et al. 2021; Du et al. 2022; Ghafari et al. 2022; Mallapaty 2022; Magiorkinis 2023; Markov et al. 2023). Several hypotheses have been proposed for the origin of VOCs, including persistent evolution in a few chronically infected COVID-19 patient (Choi et al. 2020; Rambaut, Loman, et al. 2020; Kemp et al. 2021; Hill et al. 2022; Scherer et al. 2022), cryptic circulation in a human population with insufficient samples (Wilkinson et al. 2021; Brito et al. 2022), reverse-zoonosis from animal hosts such as rodents and mink (Oude Munnink et al. 2021; Wei et al. 2021; Hale et al. 2022). Exploring the differences of selective forces in the two stages may help us understand the lack of intermediate sequences of emerging VOCs.

In this study, we track the evolution of SARS-CoV-2 in Stage I through the transition to Stage II. By comparing the evolutionary forces in the two stages, we would know whether and how the current exclusive focus on Stage II evolution may bias, or even distort, the understanding of long-term viral evolution, including the emergence of VOCs. In particular, we may need this understanding to anticipate the future of COVID-19.

## Results

**Fig. 1.**
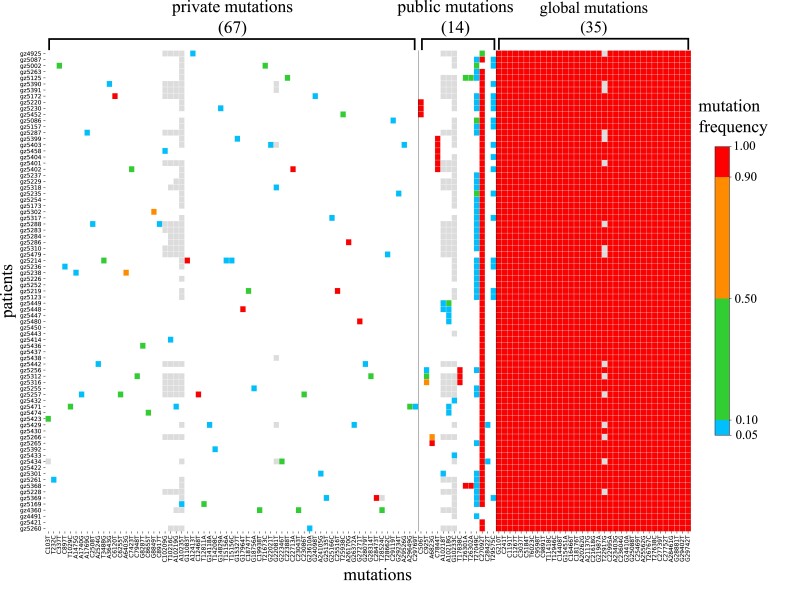
Heatmap portraying SARS-CoV-2 mutations in 79 patients. Each row is a patient's mutation profile and each column is the mutation across patients. The iSNV frequency in each host is indicated by color (gray color denoting sites with unreliable reads). The 116 mutations are classified into three groups from left to right: 67 private (one occurrence), 14 public (multiple occurrences), and 35 global (all patients) mutations. Note that public mutations are relatively rare, compared with private mutations, suggesting a hurdle of transmission for private mutations. The reference genome is Wuhan-Hu-1.

In this study, we present a data set of 79 COVID-19 confirmed cases. The mutation profile of the viral genomes within each patient, relative to the reference genome (Wuhan-Hu-1), is shown in figure 1. This dataset is uniquely informative in two ways. First, the contact records of this cohort of patients are available. Second, the viral sequences from each patient are shown as iSNVs with their frequencies indicated by color. Although fixed mutations are no longer “variants” in the strict sense of the word, they used to be iSNVs until reaching fixation. Hence, they are still classified as variants.

The 116 mutations, detected in the cohort of 79 COVID-19 patients, are classified into three groups which are, from left to right in figure 1, 67 private, 14 public, and 35 global mutations. Private mutations occur in only one single individual while public and global mutations are observed, respectively, in multiple (usually 2–10) and almost all (>70) individuals. Note that the green-to-red gradient denotes the increase in frequency with the red color showing near-fixation within the individual. Eight of these sites (four private, three public, and one global) are marked light gray. These are sites of low read depth (<100) packed in a 25 bp stretch of the genome. These gray dots should be considered uninformative sequencing reads.

It is visually obvious that global mutations are a sea of red dots. The 35 global iSNV mutations, with intrahost frequency >0.9, overlap with the defining polymorphisms of Delta strain (A23403G, C22995A) (Planas et al. 2021; Ruan, Hou, et al. 2022), thus confirming the infection by Delta strain. Importantly, red color sites are also frequently seen among private and public mutations (fig. 1). The pattern suggests that an iSNV usually has to reach a high frequency (colored red) within a few individuals before it spreads through the population. In other words, Stage II evolution commences only after the completion of Stage I. With two distinct stages of evolution, each stage can now be analyzed separately, thus simplifying the task of analyzing a complex process.

### Transmission of iSNVs From Donors to Recipients

**Fig. 2.**
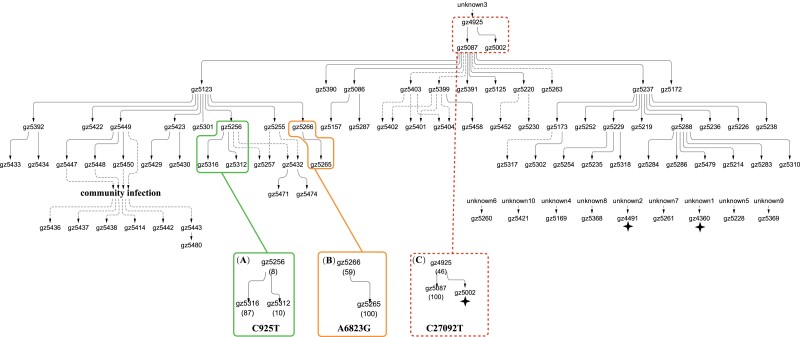
The transmission of three public mutations with mild intrahost fitness. The transmission network of 79 patients is shown in the upper panel. These three mutations (C925*T*, A6823*G*, and C27092*T*) are of moderate frequency (8%, 59%, or 46%) when first observed, but increased to higher frequency in later recipient patients. The spread of C925*T* (*A*) and A6823*G* (*B*) are limited and present in only three and two individuals, respectively. C27092*T* (*C*) reaches fixation (>95%) in all but three downstream recipients (marked by an asterisk). This mutation is deemed mildly advantageous as it has taken an unknown length of time to reach the high iSNV frequency prior to entering this cohort of patients.

**Fig. 3.**
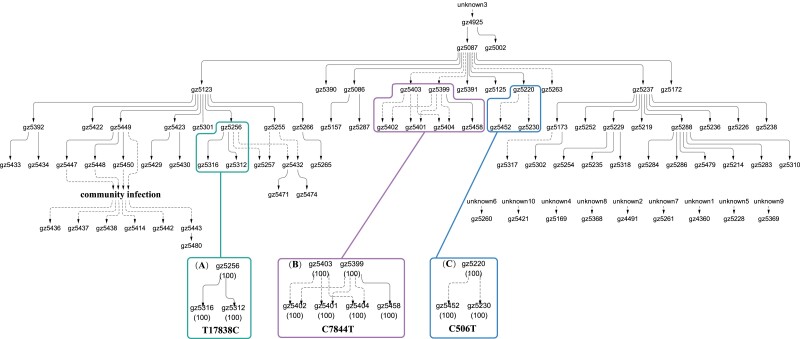
The transmission of three public mutations with strong intrahost fitness. These mutations reach 100% when first observed but are absent in the donors, thus suggesting large fitness gain in the new host. However, the spread of these mutations is limited in the cohort of patients with T17838*C* (*A*), C7844*T* (*B*), and C506*T* (*C*) present in only three, six, and three patients.

**Fig. 4.**
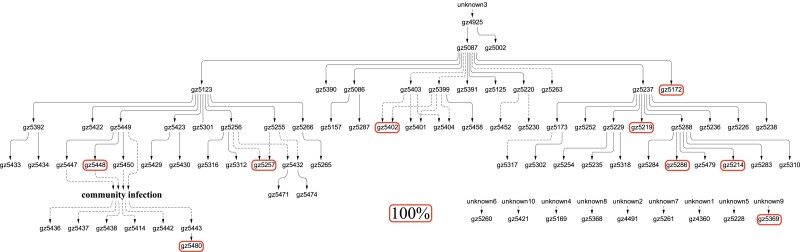
The limited spread of nine private mutation with strong intrahost fitness. Each of the nine mutations is present and, most importantly, fixed in only one host (marked by the red-border box). They are absent either upstream or downstream of this one patient, thus suggesting large fitness gain within the host but little or no transmission advantage between hosts.

The data set of figure 1 also records the detailed contact information among this cohort of 79 patients, shown in figures 2–4. The contact records establish the chain of transmission among patients (solid arrows) with some ambiguities (dotted arrows). Most important, these figures reveal the circumstances under which mutations are transmitted (becoming public) or not transmitted (remaining private).

Figures 2–4 show 15 mutations that occur in only parts of the transmission chains which are either public or private. Global mutations that occur in nearly all individuals, usually at iSNV > 0.9, are not shown. Of the three kinds, public mutations are the least abundant as they are the bridge between private and global mutations.

Public mutations have different degrees of within-host advantage, as shown in figures 2 and 3, respectively. Figure 2 displays mutations of moderate selective advantage within individuals. These are iSNV mutations that increase their frequencies step by step in more than one individual. The first one, C925T, has not reached fixation in any individual in the transmission chain. The second one, A6823G, reached fixation in the recipient from the donor (gz5266) with iSNV frequency at 59%. This iSNV seems to be a de novo mutation in gz5266 as it is not seen upstream of the transmission chain. The two mutations are deemed “moderately” advantageous within hosts only in comparison with the mutations of figures 3 and 4 below. After all, the ability to increase to a high frequency in 2–3 transmissions is impressive.

The third mutation of figure 2, C27092T, appears in the first patient (gz4925) of this cohort with the iSNV frequency of 46%. C27092T could be the weaker within-host mutation among the 15 mutations identified in this chain. We infer its weakness for two reasons. First, it is already at 46% at the beginning of the chain. Even if it rose to this frequency de novo in gz4925, it is still weaker than most others. Besides, it is likely that C27092T arose earlier and has taken some time to reach 46%. Second, C27092T failed in one of the two recipients (gz5002) from gz4925. In a mapped chain like this one, one can distinguish between nontransmission and post-transmission failure. Importantly, the box surrounding gz4925, 5002, and 5087 has dotted lines to indicate that all other patients outside of the box has C27092T at 100%. We will return to this mutation after figures 2 and 3 are presented.

The transmission patterns of figure 2 suggest that unfixed iSNVs must have a strong population structure in both space and time. In other words, samples taken at different times, or from different tissues, of the same individual would often be quite different in mutation profile (Popa et al. 2020; Gaoet al. 2021; Lythgoe et al. 2021; Ruan, Hou, et al. 2021; Li, Du, et al. 2022). Such a population structure may also explain why donors and recipients, or two recipients downstream of the same donor, often have different mutation profiles. In contrast, iSNVs reaching 100% are more often truly fixed in the host such that all samples would carry the mutation at ∼100%.

In figure 3, the three public mutations are quite different from those of figure 2. Each of the three iSNVs is a de novo mutation as it is absent upstream of the host along the transmission chain. Since each reaches 100% in the host where it is first observed, the speed of spread would suggest substantial selective advantage. With that, one might have expected the mutations to have spread widely but, instead, all of them get transmitted only once or twice. In other words, the advantage appears to be mainly within the host but does not extend to a transmission advantage between hosts.

The conjecture that the selective advantages in the two stages may be decoupled can be seen more clearly in figure 4. These are 9 de novo mutations that, like those of figure 3, rise to 100% within the host. Their further spread to other individuals, however, is completely absent. Thus, rapid rises of mutations within hosts rarely result in subsequent widespread transmission among hosts. We now return to the C27092T mutation of figure 2 which, as stated above, is the weakest iSNV within hosts. Interestingly, it is the only mutation that comes very close to being a global mutation, thus hinting its strength in transmission between hosts. In short, figures 2–4 together suggest that selection for fitness characteristics in Stage I and Stage II may be uncorrelated, or even antagonistic.

### Selection Within- versus Between-hosts—Two Uncorrelated Forces

**Fig. 5.**
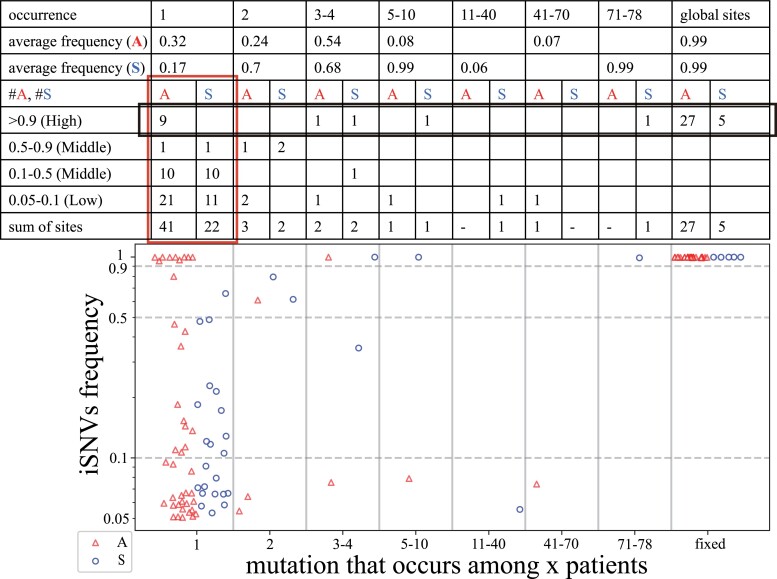
The number of nonsynonymous and synonymous mutations within and among hosts. The lower panel shows the relationship between iSNVs frequency (Y-axis) and the occurrence of iSNVs (X-axis) in 79 patients. Each nonsynonymous mutation (*A*) or synonymous mutation (*S*) is shown by a red triangle or blue circle. The upper panel calculates the number of A and S across different occurrences of iSNVs. The red-border box depicts the iSNV evolution and the black-border box depicts the evolution of high-frequency iSNVs in the human population. The A:S ratios show how positive and negative selection operate in the viral evolution (see the main text).

The total results of figures 1–4 are summarized in figure 5 with the synonymous (S) and nonsynonymous (A for amino acid altering) mutations separately tallied. To detect selection, the A:S ratio is a conventional measure (Li et al. 1985; Nei and Gojobori 1986; Yang and Nielsen 2000). If there is no selection on all mutations, the expected A:S ratio would be the same in any grouping of mutations. The neutral A:S ratio is a function of the codon usage and the nucleotide substitution pattern of each genome; for example, the A:S ratio in the human genome is ∼2.5 (Fay et al. 2001; Voight et al. 2006; Fu and Akey 2013; Martincorena et al. 2017). An observed A/S ratio larger (or smaller) than the neutral one is an indication of positive (or negative) selection for nonsynonymous changes.

Below, we first analyze the influence of selection in Stage I using private mutations, as shown in the red-border box of figure 5. We then analyze selection in Stage II, using mutations that reach iSNV frequency ≥ 0.9, as shown in the black-border box.

#### Selection for Viral Proliferation Within Hosts (The Red-border Box)

The iSNV frequencies in the red-border box of figure 5 are grouped into 3 bins, Low (L, 0.05–0.1), Middle (M, 0.1–0.9) and High (H, >0.9). Frequencies <0.05 are not used as errors below 0.05 are high. From the L to M bin, the A:S ratio decreases from 1.9 (21:11) to 1.0 (11:11). The standard population genetic interpretation (Fay and Wu 2003; Fu and Akey 2013; Wang et al. 2018; Chen et al. 2022) is that the L bin mutations consist mainly of neutral and deleterious mutations. These deleterious mutations have not been eliminated yet but will be eventually. In the M bin, with the deleterious mutations eliminated, it contains mostly neutral mutations.

In contrast, the A:S ratio increases from 11:11 to 9:0 between the M and H bin (*P* = 0.012 by Fisher's Exact Test). A salient feature of advantageous mutations is that their frequency spectrum tilts toward the high frequency bins (usually >0.8 in frequency; see (Wang et al. 2018)). It is interesting that the low-to-median frequency portion (<0.7) is not strikingly different from the neutral mutation spectrum. Hence, the high A:S ratio in the H bin is most easily explained by the spread of advantageous mutations.

#### Selection for Viral Spread Among Hosts

We now examine the interhost selection (Stage II) by examining the mutation occurrences from left to right in figure 5. We first use the last row of the table in figure 5 that sums up all iSNVs with a frequency of >0.05. If iSNVs with a frequency >0.05 are somewhat advantageous within individuals, as alluded to above, the sums should reflect the average advantage within hosts.

As shown in the table, the A/S ratio is 1.86 (41:22), 1.0 (7:7) and 5.4 (27:5) for private, public and global mutations, respectively. Generally, the A/S ratio in the population would decrease as the frequency increases, due to the working of negative selection. However, this trend may not necessarily be the expectation in viral evolution since the mutations have already been through one round of selection in Stage I. In particular, given the large number of virions within a single individual, the mutation at the time of its emergence is likely to be <10^−6^ in frequency. In that case, iSNVs of even 0.05 in frequency are likely to be somewhat advantageous. At least, it is reasonable to assume that such iSNVs are not deleterious within hosts. In short, if the selective advantages in State I and II are correlated, the decrease in the A/S ratio from low (private mutations) to medium (public mutations) frequencies reported above (1.86 to 1.0) is opposite of the expectation. In the next step from public to global mutations, the A/S ratio does increase from 1.0 (7:7) to 5.4 (27:5) as expected.

To test the postulate that the selective advantage in Stage I does not translate to an advantage in Stage II, we next focus on high-frequency iSNVs that should have the strongest advantages in Stage I (see the first row of the table with a black-border box) among all iSNVs. While we use A/S ratios to gauge the effects of selection above, the number of synonymous mutations in the iSNV > 0.9 class is too small to yield informative A/S ratios. (In fact, the paucity of such synonymous iSNVs is an indication that they are rarely advantageous within hosts to reach a high frequency.)

We therefore ask the following question: Given 9 nonsynonymous iSNVs > 0.9 that are private, how many public mutations are expected? We use the formula (Fu 1995) of *f_i_* = *θ*/*i* where *f_i_* is the number of mutations occurring in *i* of the 79 patients and *θ* is a constant for the population. Figure 5 shows *f*_1_ = *θ* = 9. Hence, the expected number of public mutations that are high frequency iSNVs should be ∑*_i_*_=2, 78_*θ*/*i* ∼ 36. It is striking that the observed number is only 1, nowhere close to the expected 36. Clearly, fixed private iSNVs are not transmitted to become public iSNVs. For a succinct summary of this section, the selective advantage as an iSNV in Stage I may be a liability in Stage II of interhost transmission.

#### Private and Global Mutations in Association With Different Viral Genes

We now ask where private and global mutations may fall among the viral genes. Public mutations are too few to be included in this analysis. We compare the S (Spike) protein with the rest of the viral genome. As shown in table 1, global mutations tend to fall in the S protein more often than expected, based on the size consideration (13% of the genome). Indeed, S protein mutations are widely known to affect viral transmission via cell attachment and entry. Interestingly, private mutations do not show an aggregation on the S protein. Perhaps, given the small number of virions that are transmitted between individuals (see the next section), the ability to be attached to cells is critical. In intrahost selection, the number of virions is so large that many other forces may be at least as important as the attachment efficiency.

**Table 1.** Numbers of iSNVs With Frequency > 0.8 (or > 0.2) From Figure 5 by Genomic Location.

	S protein (13%)	Non-S protein (87%)	Total
Private mutations	1 (1)	8 (17)	9 (18)
Global mutations	8 (8)	24 (24)	32 (32)

In summary, we ask whether the selective forces in the two stages are correlated. While the transmission patterns of figures 2–4 do not find evidence of strong correlation, figure 5 offers a more definitive answer. Whether an advantage in Stage I is advantageous, neutral or disadvantageous in Stage II would depend on how often the fitness traits in the two stages overlap. Indeed, the two types of traits may even be antagonistic (see Discussion).

### The Problem of Transmission Bottleneck *N*_0_

In this last section, we address the *N*_0_ estimation. The whole study is based on the transmission of within-host diversity from the donor to the recipients. Hence, if *N*_0_ is (or is very close to) 1, then no diversity could be transmitted. Although several studies (Braun et al. 2021; Lythgoe et al. 2021; Martin and Koelle 2021; Wang, Wang, et al. 2021; Li, Deng, et al. 2022) estimate a very tight bottleneck *N*_0_, often including *N*_0_ = 1 in the procedure, these calculations are flawed as explained below.

**Fig. 6.**
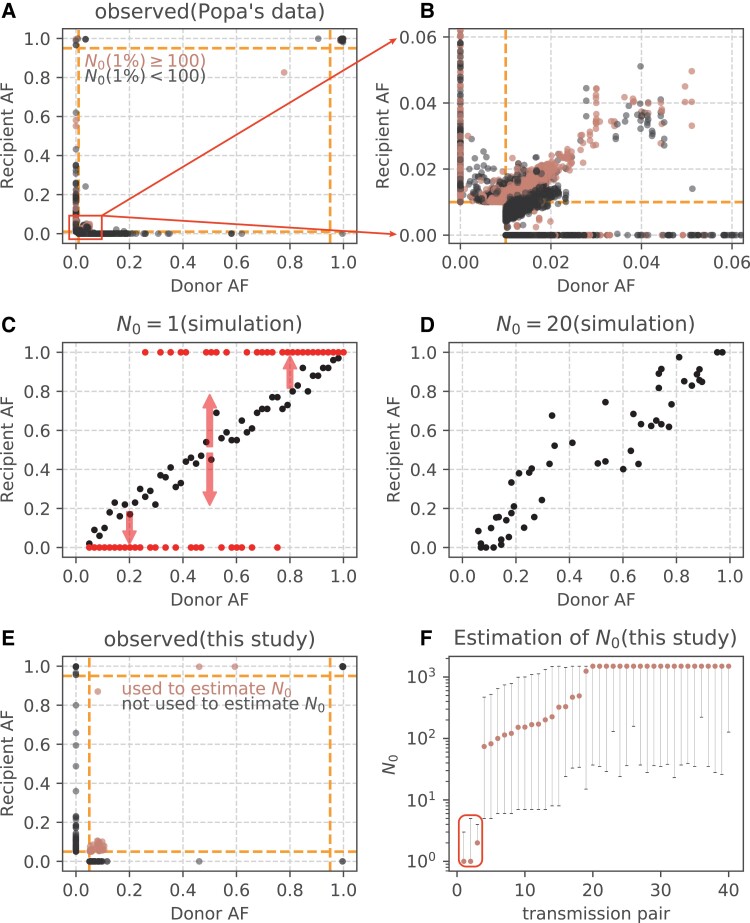
Allele frequency (AF) changes between donor and recipient used in estimating *N*_0_. (*A* and *B*) AF changes among 39 donor–recipient pairs (Popa et al.). (*B*) magnifies the low frequency portion of (*A*). (*C*) The expected AF change in donor–recipient pair when *N*_0_ = 1 (red points) or 100 (black points) based on the binomial sampling. The arrows indicate the distribution of fixed or lost variants when *N*_0_ = 1. (*D*) The change of AF when *N*_0_ = 20. (*E*) Allele frequencies of 40 donor–recipient pairs in this study. The sites used to estimate *N*_0_ are marked by orange points, which are detected in both donors and recipients. Orange dashed lines show the frequency threshold of 5%. (*F*) Estimated *N*_0_ across 40 transmission pairs. Among the 40 available pairs, the low estimates from three pairs are outliers (red-border box) due to the presence of advantageous variants (C925*T*, A6823*G*, C27092*T*). Orange points represent the maximum likelihood estimates and the error bars denote the 95% confidence interval.

Most studies use the full dataset as that of figure 6*A* and *B* (from Popa et al.), which show many sample-specific variants either on the X-axis (donor specific) or Y-axis (recipient specific). These variants most likely have emerged after, and hence not involved in, the transmission. As the de novo variants are maximally different between donor and recipient, they would yield a maximal likelihood estimate (MLE) of *N*_0_ = 1 by the binomial sampling. In such cases, MLE is simply “the best among the incorrect” as shown in figure 6*C*. The red dots represent the donor–recipient relationship that is a far *N*_0_ = 1 departure from those of figure 6*A* and *B*. As *N*_0_ increases, figure 6*D* shows the pattern of *N*_0_ = 20; if *N*_0_ = 100, the pattern is shown by the black dots of figure 6*C*.

Overall, if we factor in measurement errors in the estimation, the prudent (and conservative) estimation would be *N*_0_ ≥ 10, even if the actual *N*_0_ is 1,000. Most important, the intrahost polymorphism should be integrated into the analyses except when *N*_0_ ∼ 1, an estimate that can be convincingly rejected.

Finally, in an attempt that is not overly conservative, we estimate *N*_0_ by the beta-binomial method (Sobel Leonard et al. 2017). Sample-specific variants (i.e., variants detected only in donors or recipients) are excluded from the estimation as almost all of them are de novo mutations. Among the 40 available transmission pairs, the estimates from three pairs are outliers (the red-border box) in figure 6*F*. The low estimates, due mainly to three advantageous variants (C925T, A6823G, C27092T; see figs. 2 and 6*E*) are highly biased and should be excluded in *N*_0_ estimation. The remaining 37 pairs yield *N*_0_ estimates of 70–500 in 15 pairs and 1,200–1,500 in 22 pairs (fig. 6*F*). Our estimation is thus in agreement with the study that furnishes figure 6*A* and *B* (Popa et al. 2020) by rejecting *N*_0_ ∼ 1.

## Discussion

Any virus in the course of evolution has to move through two stages. It has to rise to a high frequency within the individual(s) to have a chance for transmission (Stage I). In Stage II, the virus has to enable the host to transmit it. We document in this study that the selective forces in the two stages are uncorrelated, and possibly antagonistic. In the extreme cases, a mutation that manages to become dominant within individuals is unable to spread in the population, or vice versa, then viral evolution simply could not proceed. We have previously reported that SARS-CoV-2 has been in a “runaway” mode that sped up its evolution greatly (Ruan, Hou, et al. 2022). This report shows that this runaway evolution may have been tempered or constrained by the two-stage evolution.

There are many reasons why selection may operate divergently within versus between hosts. For example, a mutation that causes faster viral growth in all tissues outside of the respiratory tract may be the dominant strain in the host, but this mutation could not be transmitted. On the other hand, a cold-temperature tolerant mutant that is suited to transmission may not compete well within the host. Several lines of evidence have shown that strains more competitive in the hosts often lose out to the less competitive ones in human populations. For example, Omicron is less efficient in replication and fusion compared with Delta (Zhao et al. 2022), but Omicron has displaced Delta in human populations. Also, Omicron is more infectious than Delta but has a lower viral load than Delta (Puhach et al. 2022), even in rhesus macaque (van Doremalen et al. 2022). In other cases, the trend also appears true. For example, in chronic SARS-CoV-2 infections, Kemp et al. (2021) found that a single spike mutation D796H that decreases susceptibility to neutralizing antibodies actually results in infectivity decline. A different study (Lee et al. 2023) also found that spike M1237I mutation increase viral assembly and secretion but decreases efficiency of transmission. The evidence supports the posit that selection in Stage I and Stage II may be antagonistic.

The antagonism enables the mutations that are deleterious in Stage I evolution (but generally gain fitness advantage in Stage II evolution) to persist in multiple hosts for a long time, greatly retaining the genetic diversity of virus. At the same time, many adaptive mutations would emerge during Stage I evolution, although these mutations may have no competitive advantage in Stage II evolution. Most spontaneous mutations are deleterious according to evolutionary theory (Shen et al. 2022), so there are very few mutations that are adaptive in both Stage I and Stage II evolution. However, the antagonistic pleiotropy (Williams 1957) allows the mutations, which are only partially favorable in either Stage I or Stage II evolution, to have more staying power in an evolutionary context. In this way, the virus can weigh its competitive advantages during the two stages, and finally form a VOC variant that gain overall benefit within and between hosts by possible hitchhiking or recombination.

**Fig. 7.**
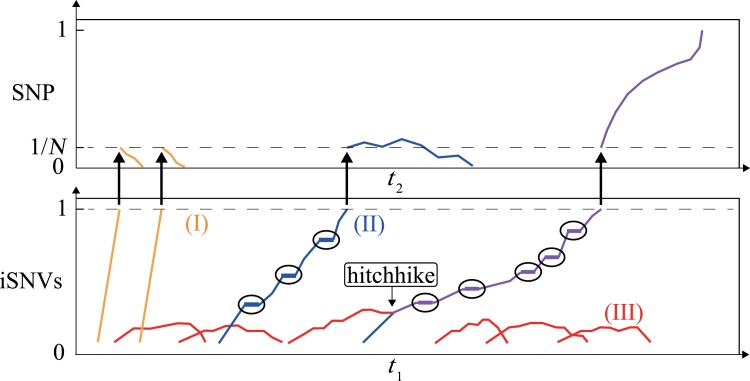
The evolutionary model of variant of concern (VOC). There are three main types of variants in the two-stage evolution. The lower and upper panels depict Stage I and Stage II evolution, respectively. Type I (yellow) has high intrahost fitness but is limited in the ability of transmission. Type II (blue) is moderately advantageous within host but slightly disadvantageous or neutral in Stage II evolution. Type III (red) gains an advantage in interhost transmission but generally cannot get out of the gate in Stage I evolution. The staircase trajectory represents the transmission between hosts, highlighted by a circle. Since it is unlikely for a single mutation to be beneficial in both stages, Type III variant may hitchhike with Type II variant to a high frequency in Stage I. At the same time, Type III variant can compensate for the transmission deficiency of Type II variant, leading to the emergence of VOC (purple line).

We hence propose a model in figure 7 where a mutant has to rise to a high frequency in Stage I (the lower panel for iSNVs) before it can enter the competition in Stage II (the upper panel for SNPs). The model incorporates three types of iSNVs as presented in Results. Type I is the mutations of figure 4 that have high fitness advantage within hosts but do not get transmitted between hosts. Type I mutations contribute little to the long-term viral evolution.

Type II iSNVs confer moderate advantages in Stage I. These mutations must increase their frequencies step by step via multiple hosts (shown by the staircase trajectory), thus requiring much longer time to become fixed iSNVs than Type I mutations. It is expected that Type II mutations accumulate continually in this slow process. We also note that even a moderate advantage in Stage I may be associated with a disadvantage in Stage II. Even with a fitness disadvantage in Stage II (basic reproductive number *R*_0_ < 1), Type II mutations could still spread among multiple hosts due to the stochasticity of early transmission but eventually become extinct in host population (Ruan, Wen, et al. 2021). Hence, only a small fraction of advantageous mutations of Type II could be established in the host population.

Type III iSNVs could confer an advantage in Stage II but few of them would realize that potential as they generally do not get out of the gate in Stage I. Occasionally, they may hitchhike with Type II mutations to a high frequency in Stage I. In reciprocity, Type III mutations can compensate for the transmission limitation of Type II mutations, eventually leading to the emergence of successful strains.

Interestingly, hitchhiking and compensation have been detected in persistent SARS-CoV-2 infection in immunosuppressed individuals (Kemp et al. 2021). The mutant D796H alluded above is a Type II mutation found in the patients. After convalescent plasma therapy, a spike deletion mutant ΔH69/ΔV70, with a higher level of infectivity, compensates for the reduced infectivity of the D796H mutation. With the double mutants of D796H and ΔH69/ΔV70, the strain became dominant in the host. Furthermore, in our study, mutation T27049C may be a Type III mutation as it occurs in 41 patients, but at low iSNV frequencies of 5–11% (supplementary fig. S1, Supplementary Material online and fig. 1). In other words, T27049C has limited within-host proliferation but appears to be good at transmission.

The model thus explains a most perplexing feature of SARS-CoV-2 evolution. Since the beginning of COVID-19, there have been six waves of viral strain, referred to as W0–W5 (Ruan, Hou, et al. 2022) where W3, W4, and W5 are, respectively, the Alpha, Delta, and Omicron wave. Each wave carries a set of mutations (21 for Alpha, 31 for Delta, and >50 for Omicron) that represent a complete replacement of those of the previous wave. Strikingly, each replacement happened in a few weeks with the sudden appearance of a new strain carrying the full set of mutations (Wei et al. 2021; Mallapaty 2022; Ruan, Hou, et al. 2022; Ruan, Wen, et al. 2022). A best documented replacement is the Alpha–Delta transition whereby Delta sweeping through within a month.

The mechanism can be explained by the model of figure 7 whereby multiple Type II and III mutations are slowly assembled into a new strain. The process happens in only a few individuals. Because the process is hardly noticeable during the assembly phase, the eventual emergence of the new strain would appear to be very sudden. This suddenness is merely a perception. Several hypotheses of VOC origins (Kemp et al. 2021; Oude Munnink et al. 2021; Wei et al. 2021; Du et al. 2022; Ghafariet al. 2022; Hill et al. 2022; Mallapaty 2022; Magiorkinis 2023; Markov et al. 2023) have been proposed to understand the emergence of VOCs, but the lack of intermediate sequences is an important obstacle to our accurate understanding of the origin of VOCs. All the five VOCs (Alpha, Beta, Gamma, Delta, and Omicron) had evolved from the pre-VOC progenitors, rather than from one another (Carabelli et al. 2023), suggesting the undetected lineages could be evolving for a long time. These pre-VOCs may be largely noncompeting and likely occupy semi-independent epidemiological niches that are not regionally defined (Mutz et al. 2022). An uncorrelated, and possibly antagonistic driving forces in Stage I and Stage II evolution, found in this study, provide a new and proper explanation for the lack of intermediate sequences and the possible emergence pattern of VOCs.

Long before Delta became prevalent, most (27) of the 31 Delta mutations are already present in very low frequency in India (Ruan, Hou, et al. 2022). Unlike typical natural populations whereby such rare mutations are scattered across haplotypes with each harboring 1–2 such mutations, ALL 27 rare mutations are found on the same, albeit rare, haplotype. Importantly, although a rare haplotype can be quickly lost in most evolutionary processes, such a rare viral strain would not be lost in the population due to its intrahost advantage, stated explicitly in figure 7. The sudden appearance has at times meant the existence of animal reservoirs in the literature (Oude Munnink et al. 2021; Wei et al. 2021; Mallapaty 2022). For example, Wei et al. (2021) have suggested that Omicron was assembled in mice before it jumped to humans. Such an explanation has its limitation because Delta, as well as other new strains, also experienced the swift replacement but these events are still believed to have evolved solely in humans.

The transmission bottleneck of SARS-CoV-2 is a controversial issue (Popa et al. 2020; Armero et al. 2021; Braun et al. 2021; Lythgoe et al. 2021; Martin and Koelle 2021; Li, Deng, et al. 2022; Li, Du, et al. 2022). Our analysis suggests that *N*_0_ has been severely underestimated, mainly because the genetic divergence between donor and recipient is exaggerated. While it is true that “the larger the divergence, the smaller the *N*_0_ estimate”, small *N*_0_ in fact does not lead to the divergence actually observed. The divergence between donor and recipient is often the results of de novo mutations that fall on the X and Y axes of figure 6. Even *N*_0_ = 1 could not account for the divergence. In some cases, a few advantageous mutations may also bias the *N*_0_ estimate downward whereas small *N*_0_ should affect *all* mutations. As in some other reports (Popaet al. 2020), our analyses show *N*_0_ to be at least in the hundreds and large enough to transmit the genetic diversity between hosts.

In this context, a key question about COVID-19 3 years after its onset is whether Omicron is the last wave. While subvariant VOCs of Omicron are common, the threat would come from a new wave of variants that shares no mutations with Omicron. It is not farfetched that Delta may re-emerge from the ashes as Delta has not entirely disappeared (Yaniv et al. 2022). The re-emergence of a previous wave has been reported; for example, Wave 1 of Ruan, Hou, et al. (2022) disappeared after W2 but later re-emerged as W3 (Alpha) after the acquisition of additional mutations. The monitoring of VOCs should include features of figure 7 by focusing on potential new waves in addition to new subvariants of Omicron. In conclusion, Stage I appears to exert a strong selective pressure on SARS-CoV-2 as it filters out many mutations and deprive them the opportunity to compete in Stage II. This stage of evolution has been neglected in previous studies and deserves a lot more attentions.

## Materials and Methods

### Samples and Transmission Network

Our study included 79 COVID-19 patients infected with SARS-CoV-2 Delta strain admitted in the Guangzhou Eighth People's Hospital from May 21 to June 18, 2021. All patients of this cohort were confirmed by the local Centers for Disease Control and transferred to Guangzhou Eighth People's Hospital, Guangzhou. Epidemiological data were collected including the exposure histories directly to the confirmed cases (see supplementary table S2, Supplementary Material online). Transmission chains are visualized by Cytoscape v3.9.1 (Shannon et al. 2003).

### Viral RNA Sequencing

The sequencing library was prepared using an amplicon-based enrichment method as described previously (Wang, Chen, et al. 2021). All samples were sequenced on the MGISEQ-2000 platform.

### iSNVs Calling

1) The raw sequencing data were first quality controlled using fastp v0.12.4 (Chen et al. 2018) to trim adapters and cut low-quality bases (quality scores < 20). The qualified reads were sent to trim PCR primers by cutadapt v4.1 (Martin 2011). 2) Sequencing reads were then pair-ended aligned to the reference genome sequence (Wuhan-Hu-1, GenBank accession no. NC 045512.2; Wu et al. 2020) using BWA v0.7.17 (Li and Durbin 2009). The bam files were sorted by SAMtools v1.15.1 (Li et al. 2009) and primers were further trimmed by iVar v1.3.1 (Grubaugh et al. 2019). 3) We identified iSNVs relative to reference genome using the following criteria: alternative allele support depth ≥ 10, total depth ≥ 100, iSNVs frequency ≥ 5%, iVar PASS = TRUE. 4) After calling variants, we used ANNOVAR software (Yang and Wang 2015) to annotate the variants and found the count of alternative allele and total depth for each variant using SAMtools. We identified a total of 116 mutations, including 67 private, 14 public, and 35 global mutations (see supplementary table S1, Supplementary Material online).

### Reanalysis of Previously Published SARS-CoV-2 Data

We reanalyzed 138 COVID-19 samples with clinical information of Popa's data (Popa et al. 2020), which including 39 transmission pairs. We downloaded the clinical information and vcf files available at https://doi.org/10.5281/zenodo.5224640. We used python scripts to merge the frequency of iSNVs of these 138 samples. For each transmission pair, we identified the variants at frequency of ≥1% and showed the allele frequency change between donor and recipient. We used the threshold 100 of transmission bottleneck (*N*_0_), estimated by Martin and Koelle (2021), to divide the alleles into two groups.

### Calculating the Number of Nonsynonymous (*N*) and Synonymous Sites (*S*) in SARS-CoV-2 Reference Genome

We downloaded 12 coding region sequences (CDSs) of SARS-CoV-2 reference genome (Wuhan-Hu-1, GenBank accession no. NC_045512.2) from NCBI, including ORF1ab, ORF1a, S, ORF3a, E, M, ORF6, ORF7a, ORF7b, ORF8, N, and ORF10. We removed the stop codons of all the 12 CDSs first. Production of pp1ab depends on the occurrence of a −1 programed ribosomal frameshift at nucleotide 13,468, just four codons upstream of the ORF1a (266–13,483) termination codon. After cutting the overlapping segments (nucleotides 266–13,468) between ORF1ab and ORF1a from ORF1a, we concatenated the trimmed ORF1a with the remaining 11 CDSs (including ORF1ab) into a single sequence (29,244 nucleotides in total). YN00 from PAML v4.9a (Yang 2007) was then used to calculate the *N* (the number of nonsynonymous sites) and *S* (the number of synonymous sites). There are 22,599.3 nonsynonymous (*N*) and 6,644.7 synonymous (*S*) sites in the coding regions of the reference genome. Thus, with no selection, the A/S ratio should be close to 3.4 (22,599.3/6,644.7).

### Genetic Drift in a Growing Population

Based on branching process, Chen et al. (2017) obtained the genetic drift after single generation. Here, we expand it and get the genetic drift after multiple generations, which can be used to estimate the variance of alternative allele frequency within host. According to Chen et al. (2017), the average and variance of population size at time *t* are


$$E(N_{t})=N_{0}E{\left(k\right)}^{t}$$

$$V(N_{t})=\{\begin{array}{ll} N_{0}V(k)t, & \mathrm{if}\;E(k)=1\\ N_{0}V(k)E{\left(k\right)}^{t-1}\frac{E{\left(k\right)}^{t}-1}{E(k)-1}, & \mathrm{if}\;E(k)>1. \end{array}$$

Assuming there are two kinds of alleles, and their numbers at generation *t* are *I_t_*, *J_t_*. *I_t_* and *J_t_* will be independent. If there is no selection,


(*A*1) $$E(\;p_{t})=E\left(\frac{I_{t}}{I_{t}+J_{t}}\right)=p=\frac{I_{0}}{N_{0}}\;$$

(*A*2) $$V(\;p_{t})=V\left(\frac{I_{t}}{I_{t}+J_{t}}\right).$$

According to bivariate first order Taylor expansion (Duris et al. 2018), when *E*(*k*) > 1


(*A*3) $$\begin{array}{l} V(\;p_{t})=V\left(\frac{I_{t}}{I_{t}+J_{t}}\right)\approx \frac{E{\left(I_{t}\right)}^{2}}{E{\left(N_{t}\right)}^{2}}\left[\frac{V(I_{t})}{E{\left(I_{t}\right)}^{2}}-2\frac{\mathrm{cov}(I_{t},N_{t})}{E(I_{t})E(N_{t})}+\frac{V(N_{t})}{E{\left(N_{t}\right)}^{2}}\right]\\ \;\;\;\;\;=\;p^{2}\left[\frac{I_{0}V(k)E{\left(k\right)}^{t-1}\frac{E{\left(k\right)}^{t}-1}{E(k)-1}}{{\left[I_{0}E{\left(k\right)}^{t}\right]}^{2}}-2\frac{I_{0}V(k)E{\left(k\right)}^{t-1}\frac{E{\left(k\right)}^{t}-1}{E(k)-1}}{I_{0}E{\left(k\right)}^{t}N_{0}E{\left(k\right)}^{t}}+\frac{N_{0}V(k)E{\left(k\right)}^{t-1}\frac{E{\left(k\right)}^{t}-1}{E(k)-1}}{{\left[N_{0}E{\left(k\right)}^{t}\right]}^{2}}\right]\\ \;\;\;\;\;\;=\frac{\;p(1-p)}{E(N_{t})}\frac{V(k)}{E(k)}\frac{E{\left(k\right)}^{t}-1}{E(k)-1}. \end{array}$$

Specially, when *t* = 1,


$$V(\;p_{t=1})=\frac{\;p(1-p)}{N_{0}}\frac{V(k)}{E{\left(k\right)}^{2}}$$

which is the same as eq. (5) in Chen et al. (2017).

### Simulating the Expected Allele Frequency After Transmission Basing on Branching Process Model

Assuming there are *n* alleles with corresponding frequencies $x_{1}$, $x_{2}$, …, $x_{n}$ in donor, we will obtain the expected allele frequency of recipient under a particular transmission bottleneck size *N*_0_ as follows. For traditional WF model, each allele is independent and its allele frequency in next generation will follow binomial distribution. Thus, given transmission bottleneck size *N*_0_, for the allele with frequency $x_{i}$ in donor, its frequency in donor, $x_{i}^{\prime }$, will be sampled from binomial distribution.


$$x_{i}^{\prime }∼Bin(N_{0},x_{i})/N_{0}.$$

After transmission, we assume the virus will grow to a particular number, *N_t_*, before it be sampled and sequenced. During the branching process of virus growth, we assume each virus will generate *k* number of offspring, where *k* follows a negative binomial distribution with mean *E*(*k*) and variance *V*(*k*). Thus, the expected time at which the virus is sampled to determine the recipient allele frequency (denoted by *x_t_*) is


$$t=\frac{\log(N_{t}/N_{0})}{\log(E(k))}.$$

According to eq. (A1) and eq. (A3), given the initial allele frequency $x_{i}^{\prime }$, we can obtain the mean and variance of *x_t_* when population size grows from *N*_0_ to *N_t_*:


$$E(x_{t})=x_{i}^{\prime }$$

$$V(x_{t})=\frac{x_{i}^{\prime }(1-x_{i}^{\prime })}{N_{t}}\frac{V(k)}{E(k)}\frac{E{\left(k\right)}^{t}-1}{E(k)-1}.$$

Simply, we can assume *x_t_* follows a normal distribution with mean and variance to be *E*(*x_t_*) and *V*(*x_t_*). Now, we can obtain the expected allele frequency in donor–recipient by sampling from the normal distribution.

## Supplementary Material

msad204_Supplementary_Data

## Data Availability

Raw sequencing reads have been deposited to National Genomics Data Center (https://bigd.big.ac.cn/) with submission number CRA012327 (https://ngdc.cncb.ac.cn/gsa/browse/CRA012327). And the code for calling mutations can be found in the GitHub repository GZ521_sars2 available at https://github.com/yongsen-ruan/GZ521_sars2.

## References

[msad204-B1] Armero A , Berthet N, Avarre J-C. 2021. Intra-host diversity of SARS-Cov-2 should not be neglected: case of the State of Victoria, Australia. Viruses 13:133. 33477885 10.3390/v13010133 PMC7833370

[msad204-B2] Braun KM , Moreno GK, Halfmann PJ, Hodcroft EB, Baker DA, Boehm EC, Weiler AM, Haj AK, Hatta M, Chiba S, et al 2021. Transmission of SARS-CoV-2 in domestic cats imposes a narrow bottleneck. PLoS Pathog. 17:e1009373. 33635912 10.1371/journal.ppat.1009373 PMC7946358

[msad204-B3] Brito AF , Semenova E, Dudas G, Hassler GW, Kalinich CC, Kraemer MUG, Ho J, Tegally H, Githinji G, Agoti CN, et al 2022. Global disparities in SARS-CoV-2 genomic surveillance. Nat Commun. 13:7003. 36385137 10.1038/s41467-022-33713-y PMC9667854

[msad204-B4] Carabelli AM , Peacock TP, Thorne LG, Harvey WT, Hughes J, COVID-19 Genomics UK Consortium, Peacock SJ, Barclay WS, de Silva TI, Towers GJ, et al 2023. SARS-CoV-2 variant biology: immune escape, transmission and fitness. Nat Rev Microbiol. 21:162–177. 36653446 10.1038/s41579-022-00841-7 PMC9847462

[msad204-B5] Chen Y , Tong D, Wu C-I. 2017. A new formulation of random genetic drift and its application to the evolution of cell populations. Mol Biol Evol. 34:2057–2064. 28525580 10.1093/molbev/msx161

[msad204-B6] Chen Q , Yang H, Feng X, Chen Q, Shi S, Wu C-I, He Z. 2022. Two decades of suspect evidence for adaptive molecular evolution-negative selection confounding positive-selection signals. Natl Sci Rev. 9:nwab217. 35663241 10.1093/nsr/nwab217 PMC9154339

[msad204-B7] Chen S , Zhou Y, Chen Y, Gu J. 2018. Fastp: an ultra-fast all-in-one FASTQ preprocessor. Bioinformatics 34:i884–i890. 30423086 10.1093/bioinformatics/bty560 PMC6129281

[msad204-B8] Choi B , Choudhary MC, Regan J, Sparks JA, Padera RF, Qiu X, Solomon IH, Kuo HH, Boucau J, Bowman K, et al 2020. Persistence and evolution of SARS-CoV-2 in an immunocompromised host. N Engl J Med. 383:2291–2293. 33176080 10.1056/NEJMc2031364 PMC7673303

[msad204-B9] Dellicour S , Durkin K, Hong SL, Vanmechelen B, Marti-Carreras J, Gill MS, Meex C, Bontems S, Andre E, Gilbert M, et al 2021. A phylodynamic workflow to rapidly gain insights into the dispersal history and dynamics of SARS-CoV-2 lineages. Mol Biol Evol. 38:1608–1613. 33316043 10.1093/molbev/msaa284 PMC7665608

[msad204-B10] Du P , Gao GF, Wang Q. 2022. The mysterious origins of the Omicron variant of SARS-CoV-2. Innovation (Camb) 3:100206. 35043101 10.1016/j.xinn.2022.100206 PMC8757324

[msad204-B11] Duris F , Gazdarica J, Gazdaricova I, Strieskova L, Budis J, Turna J, Szemes T. 2018. Mean and variance of ratios of proportions from categories of a multinomial distribution. J Stat Distrib Appl. 5:2.

[msad204-B12] Fay JC , Wu C-I. 2003. Sequence divergence, functional constraint, and selection in protein evolution. Annu Rev Genomics Hum Genet. 4:213–235. 14527302 10.1146/annurev.genom.4.020303.162528

[msad204-B13] Fay JC , Wyckoff GJ, Wu C-I. 2001. Positive and negative selection on the human genome. Genetics 158:1227–1234. 11454770 10.1093/genetics/158.3.1227 PMC1461725

[msad204-B14] Fu YX . 1995. Statistical properties of segregating sites. Theor Popul Biol. 48:172–197. 7482370 10.1006/tpbi.1995.1025

[msad204-B15] Fu W , Akey JM. 2013. Selection and adaptation in the human genome. Annu Rev Genomics Hum Genet. 14:467–489. 23834317 10.1146/annurev-genom-091212-153509

[msad204-B16] Gao R , Zu W, Liu Y, Li J, Li Z, Wen Y, Wang H, Yuan J, Cheng L, Zhang S, et al 2021. Quasispecies of SARS-CoV-2 revealed by single nucleotide polymorphisms (SNPs) analysis. Virulence 12:1209–1226. 34030593 10.1080/21505594.2021.1911477 PMC8158041

[msad204-B17] Ghafari M , Liu Q, Dhillon A, Katzourakis A, Weissman DB. 2022. Investigating the evolutionary origins of the first three SARS-CoV-2 variants of concern. Front Virol. 2:76.

[msad204-B18] Grubaugh ND , Gangavarapu K, Quick J, Matteson NL, De Jesus JG, Main BJ, Tan AL, Paul LM, Brackney DE, Grewal S, et al 2019. An amplicon-based sequencing framework for accurately measuring intrahost virus diversity using PrimalSeq and iVar. Genome Biol. 20:8. 30621750 10.1186/s13059-018-1618-7 PMC6325816

[msad204-B19] Hale VL , Dennis PM, McBride DS, Nolting JM, Madden C, Huey D, Ehrlich M, Grieser J, Winston J, Lombardi D, et al 2022. SARS-CoV-2 infection in free-ranging white-tailed deer. Nature 602:481–486. 34942632 10.1038/s41586-021-04353-x PMC8857059

[msad204-B20] Hill V , Du Plessis L, Peacock TP, Aggarwal D, Colquhoun R, Carabelli AM, Ellaby N, Gallagher E, Groves N, Jackson B, et al 2022. The origins and molecular evolution of SARS-CoV-2 lineage B.1.1.7 in the UK. Virus Evol. 8:veac080. 36533153 10.1093/ve/veac080 PMC9752794

[msad204-B21] Kemp SA , Collier DA, Datir RP, Ferreira I, Gayed S, Jahun A, Hosmillo M, Rees-Spear C, Mlcochova P, Lumb IU, et al 2021. SARS-CoV-2 evolution during treatment of chronic infection. Nature 592:277–282. 33545711 10.1038/s41586-021-03291-y PMC7610568

[msad204-B22] Killingley B , Mann AJ, Kalinova M, Boyers A, Goonawardane N, Zhou J, Lindsell K, Hare SS, Brown J, Frise R, et al 2022. Safety, tolerability and viral kinetics during SARS-CoV-2 human challenge in young adults. Nat Med. 28:1031–1041. 35361992 10.1038/s41591-022-01780-9

[msad204-B23] Korber B , Fischer WM, Gnanakaran S, Yoon H, Theiler J, Abfalterer W, Hengartner N, Giorgi EE, Bhattacharya T, Foley B, et al 2020. Tracking changes in SARS-CoV-2 spike: evidence that D614G increases infectivity of the COVID-19 virus. Cell 182:812–827.e19. 32697968 10.1016/j.cell.2020.06.043 PMC7332439

[msad204-B24] Lee D-C , Tai J-H, Lin H-F, Chao T-L, Ruan Y, Cheng Y-W, Chou Y-C, Lin Y-Y, Chang S-Y, Chen P-J, et al 2023. Antagonistic pleiotropy plays an important role in governing the evolution and genetic diversity of SARS-CoV-2. bioRxiv:2023.2002.2010.527437, doi:10.1101/2023.02.10.527437

[msad204-B25] Li W-H . 1997. Molecular evolution. Sunderland: Sinauer Associates Incorporated.

[msad204-B26] Li B , Deng A, Li K, Hu Y, Li Z, Shi Y, Xiong Q, Liu Z, Guo Q, Zou L, et al 2022. Viral infection and transmission in a large, well-traced outbreak caused by the SARS-CoV-2 Delta variant. Nat Commun. 13:460. 35075154 10.1038/s41467-022-28089-y PMC8786931

[msad204-B27] Li J , Du P, Yang L, Zhang J, Song C, Chen D, Song Y, Ding N, Hua M, Han K, et al 2022. Two-step fitness selection for intra-host variations in SARS-CoV-2. Cell Rep. 38:110205. 34982968 10.1016/j.celrep.2021.110205 PMC8674508

[msad204-B28] Li H , Durbin R. 2009. Fast and accurate short read alignment with Burrows-Wheeler transform. Bioinformatics 25:1754–1760. 19451168 10.1093/bioinformatics/btp324 PMC2705234

[msad204-B29] Li H , Handsaker B, Wysoker A, Fennell T, Ruan J, Homer N, Marth G, Abecasis G, Durbin R; 1000 Genome Project Data Processing Subgroup. 2009. The sequence alignment/map format and SAMtools. Bioinformatics 25:2078–2079. 19505943 10.1093/bioinformatics/btp352 PMC2723002

[msad204-B30] Li WH , Wu CI, Luo CC. 1985. A new method for estimating synonymous and nonsynonymous rates of nucleotide substitution considering the relative likelihood of nucleotide and codon changes. Mol Biol Evol. 2:150–174. 3916709 10.1093/oxfordjournals.molbev.a040343

[msad204-B31] Lythgoe KA , Hall M, Ferretti L, de Cesare M, MacIntyre-Cockett G, Trebes A, Andersson M, Otecko N, Wise EL, Moore N, et al 2021. SARS-CoV-2 within-host diversity and transmission. Science 372:eabg0821. 33688063 10.1126/science.abg0821 PMC8128293

[msad204-B32] Magiorkinis G . 2023. On the evolution of SARS-CoV-2 and the emergence of variants of concern. Trends Microbiol. 31:5–8. 36344310 10.1016/j.tim.2022.10.008 PMC9595369

[msad204-B33] Mallapaty S . 2022. Where did Omicron come from? Three key theories. Nature 602:26–28. 35091701 10.1038/d41586-022-00215-2

[msad204-B34] Markov PV , Ghafari M, Beer M, Lythgoe K, Simmonds P, Stilianakis NI, Katzourakis A. 2023. The evolution of SARS-CoV-2. Nat Rev Microbiol. 21:361–379. 37020110 10.1038/s41579-023-00878-2

[msad204-B35] Martin M . 2011. Cutadapt removes adapter sequences from high-throughput sequencing reads. EMBnet.journal 17:3.

[msad204-B36] Martin MA , Koelle K. 2021. Comment on “Genomic epidemiology of superspreading events in Austria reveals mutational dynamics and transmission properties of SARS-CoV-2”. Sci Transl Med. 13:eabh1803. 34705523 10.1126/scitranslmed.abh1803 PMC9301528

[msad204-B37] Martincorena I , Raine KM, Gerstung M, Dawson KJ, Haase K, Van Loo P, Davies H, Stratton MR, Campbell PJ. 2017. Universal patterns of selection in cancer and somatic tissues. Cell 171:1029–1041.e21. 29056346 10.1016/j.cell.2017.09.042 PMC5720395

[msad204-B38] Mutz P , Rochman ND, Wolf YI, Faure G, Zhang F, Koonin EV. 2022. Human pathogenic RNA viruses establish noncompeting lineages by occupying independent niches. Proc Natl Acad Sci U S A. 119:e2121335119. 10.1073/pnas.2121335119 PMC9191635 35639694

[msad204-B39] Nei M , Gojobori T. 1986. Simple methods for estimating the numbers of synonymous and nonsynonymous nucleotide substitutions. Mol Biol Evol. 3:418–426. 3444411 10.1093/oxfordjournals.molbev.a040410

[msad204-B40] Oude Munnink BB , Sikkema RS, Nieuwenhuijse DF, Molenaar RJ, Munger E, Molenkamp R, van der Spek A, Tolsma P, Rietveld A, Brouwer M, et al 2021. Transmission of SARS-CoV-2 on mink farms between humans and mink and back to humans. Science 371:172–177. 33172935 10.1126/science.abe5901 PMC7857398

[msad204-B41] Planas D , Veyer D, Baidaliuk A, Staropoli I, Guivel-Benhassine F, Rajah MM, Planchais C, Porrot F, Robillard N, Puech J, et al 2021. Reduced sensitivity of SARS-CoV-2 variant delta to antibody neutralization. Nature 596:276–280. 34237773 10.1038/s41586-021-03777-9

[msad204-B42] Popa A , Genger JW, Nicholson MD, Penz T, Schmid D, Aberle SW, Agerer B, Lercher A, Endler L, Colaco H, et al 2020. Genomic epidemiology of superspreading events in Austria reveals mutational dynamics and transmission properties of SARS-CoV-2. Sci Transl Med. 12:eabe2555. 33229462 10.1126/scitranslmed.abe2555 PMC7857414

[msad204-B43] Puhach O , Adea K, Hulo N, Sattonnet P, Genecand C, Iten A, Jacquerioz F, Kaiser L, Vetter P, Eckerle I, et al 2022. Infectious viral load in unvaccinated and vaccinated individuals infected with ancestral, Delta or Omicron SARS-CoV-2. Nat Med. 28:1491–1500. 35395151 10.1038/s41591-022-01816-0

[msad204-B44] Rambaut A , Holmes EC, O’Toole A, Hill V, McCrone JT, Ruis C, du Plessis L, Pybus OG. 2020. A dynamic nomenclature proposal for SARS-CoV-2 lineages to assist genomic epidemiology. Nat Microbiol. 5:1403–1407. 32669681 10.1038/s41564-020-0770-5 PMC7610519

[msad204-B45] Rambaut A , Loman N, Pybus O, Barclay W, Barrett J, Carabelli A, Connor T, Peacock T, Robertson DL, Volz E. 2020. Preliminary genomic characterisation of an emergent SARS-CoV-2 lineage in the UK defined by a novel set of spike mutations. Virological. https://virological.org/t/563.

[msad204-B46] Ruan Y , Hou M, Li J, Song Y, Wang H-YI, He X, Zeng H, Lu J, Wen H, Chen C, et al 2021. One viral sequence for each host? – The neglected within-host diversity as the main stage of SARS-CoV-2 evolution. bioRxiv:2021.2006.2021.449205, doi:10.1101/2021.06.21.449205

[msad204-B47] Ruan Y , Hou M, Tang X, He X, Lu X, Lu J, Wu C-I, Wen H. 2022. The runaway evolution of SARS-CoV-2 leading to the highly evolved Delta strain. Mol Biol Evol. 39:msac046. 35234869 10.1093/molbev/msac046 PMC8903489

[msad204-B48] Ruan Y , Luo Z, Tang X, Li G, Wen H, He X, Lu X, Lu J, Wu C-I. 2021. On the founder effect in COVID-19 outbreaks: how many infected travelers may have started them all? Natl Sci Rev. 8:nwaa246. 34676089 10.1093/nsr/nwaa246 PMC7543514

[msad204-B49] Ruan Y , Wen H, He X, Wu C-I. 2021. A theoretical exploration of the origin and early evolution of a pandemic. Sci Bull (Beijing). 66:1022–1029. 33520335 10.1016/j.scib.2020.12.020 PMC7831721

[msad204-B50] Ruan Y , Wen H, Hou M, He Z, Lu X, Xue Y, He X, Zhang YP, Wu C-I. 2022. The twin-beginnings of COVID-19 in Asia and Europe-one prevails quickly. Natl Sci Rev. 9:nwab223. 35497643 10.1093/nsr/nwab223 PMC9046579

[msad204-B51] Scherer EM , Babiker A, Adelman MW, Allman B, Key A, Kleinhenz JM, Langsjoen RM, Nguyen PV, Onyechi I, Sherman JD, et al 2022. SARS-CoV-2 evolution and immune escape in immunocompromised patients. N Engl J Med. 386:2436–2438. 35675197 10.1056/NEJMc2202861 PMC9202319

[msad204-B52] Shannon P , Markiel A, Ozier O, Baliga NS, Wang JT, Ramage D, Amin N, Schwikowski B, Ideker T. 2003. Cytoscape: a software environment for integrated models of biomolecular interaction networks. Genome Res. 13:2498–2504. 14597658 10.1101/gr.1239303 PMC403769

[msad204-B53] Shen X , Song S, Li C, Zhang J. 2022. Synonymous mutations in representative yeast genes are mostly strongly non-neutral. Nature 606:725–731. 35676473 10.1038/s41586-022-04823-w PMC9650438

[msad204-B54] Sobel Leonard A , Weissman DB, Greenbaum B, Ghedin E, Koelle K. 2017. Transmission bottleneck size estimation from pathogen deep-sequencing data, with an application to human influenza A virus. J Virol. 91:e00171-00117. 28468874 10.1128/JVI.00171-17 PMC5487570

[msad204-B55] Tang X , Wu C, Li X, Song Y, Yao X, Wu X, Duan Y, Zhang H, Wang Y, Qian Z, et al 2020. On the origin and continuing evolution of SARS-CoV-2. Natl Sci Rev. 7:1012–1023. 34676127 10.1093/nsr/nwaa036 PMC7107875

[msad204-B56] van Doremalen N , Singh M, Saturday TA, Yinda CK, Perez-Perez L, Bohler WF, Weishampel ZA, Lewis M, Schulz JE, Williamson BN, et al 2022. SARS-CoV-2 Omicron BA.1 and BA.2 are attenuated in rhesus macaques as compared to delta. Sci Adv. 8:eade1860. 36399566 10.1126/sciadv.ade1860 PMC9674298

[msad204-B57] Voight BF , Kudaravalli S, Wen X, Pritchard JK. 2006. A map of recent positive selection in the human genome. PLoS Biol. 4:e72. 16494531 10.1371/journal.pbio.0040072 PMC1382018

[msad204-B58] Wang Y , Chen R, Hu F, Lan Y, Yang Z, Zhan C, Shi J, Deng X, Jiang M, Zhong S, et al 2021. Transmission, viral kinetics and clinical characteristics of the emergent SARS-CoV-2 delta VOC in Guangzhou, China. EClinicalMedicine 40:101129. 34541481 10.1016/j.eclinm.2021.101129 PMC8435265

[msad204-B59] Wang H-Y , Chen Y, Tong D, Ling S, Hu Z, Tao Y, Lu X, Wu C-I. 2018. Is the evolution in tumors Darwinian or non-Darwinian? Natl Sci Rev. 5:15–17.

[msad204-B60] Wang D , Wang Y, Sun W, Zhang L, Ji J, Zhang Z, Cheng X, Li Y, Xiao F, Zhu A, et al 2021. Population bottlenecks and intra-host evolution during human-to-human transmission of SARS-CoV-2. Front Med (Lausanne). 8:585358. 33659260 10.3389/fmed.2021.585358 PMC7917136

[msad204-B61] Wei C , Shan K-J, Wang W, Zhang S, Huan Q, Qian W. 2021. Evidence for a mouse origin of the SARS-CoV-2 Omicron variant. J Genet Genomics. 48:1111–1121. 34954396 10.1016/j.jgg.2021.12.003 PMC8702434

[msad204-B62] WHO . 2022. SARS-CoV-2 variants of concern and variants of interest. World Health Organization. Available from: https://www.who.int/en/activities/tracking-SARS-CoV-2-variants/

[msad204-B63] Wilkinson E , Giovanetti M, Tegally H, San JE, Lessells R, Cuadros D, Martin DP, Rasmussen DA, Zekri AN, Sangare AK, et al 2021. A year of genomic surveillance reveals how the SARS-CoV-2 pandemic unfolded in Africa. Science 374:423–431. 34672751 10.1126/science.abj4336 PMC7613315

[msad204-B64] Williams GC . 1957. Pleiotropy, natural selection, and the evolution of senescence. Evolution 11:398–411.

[msad204-B65] Wu C-I , Wen H, Lu J, Su X-D, Hughes AC, Zhai W, Chen C, Chen H, Li M, Song S, et al 2021. On the origin of SARS-CoV-2-the blind watchmaker argument. Sci China Life Sci. 64:1560–1563. 34269976 10.1007/s11427-021-1972-1 PMC8284035

[msad204-B66] Wu F , Zhao S, Yu B, Chen Y-M, Wang W, Song Z-G, Hu Y, Tao Z-W, Tian J-H, Pei Y-Y, et al 2020. A new coronavirus associated with human respiratory disease in China. Nature 579:265–269. 32015508 10.1038/s41586-020-2008-3 PMC7094943

[msad204-B67] Yang Z . 2007. PAML 4: phylogenetic analysis by maximum likelihood. Mol Biol Evol. 24:1586–1591. 17483113 10.1093/molbev/msm088

[msad204-B68] Yang Z , Nielsen R. 2000. Estimating synonymous and nonsynonymous substitution rates under realistic evolutionary models. Mol Biol Evol. 17:32–43. 10666704 10.1093/oxfordjournals.molbev.a026236

[msad204-B69] Yang H , Wang K. 2015. Genomic variant annotation and prioritization with ANNOVAR and wANNOVAR. Nat Protoc. 10:1556–1566. 26379229 10.1038/nprot.2015.105 PMC4718734

[msad204-B70] Yaniv K , Ozer E, Shagan M, Paitan Y, Granek R, Kushmaro A. 2022. Managing an evolving pandemic: cryptic circulation of the delta variant during the Omicron rise. Sci Total Environ. 836:155599. 35504376 10.1016/j.scitotenv.2022.155599 PMC9055682

[msad204-B71] Zeng HL , Dichio V, Rodriguez Horta E, Thorell K, Aurell E. 2020. Global analysis of more than 50,000 SARS-CoV-2 genomes reveals epistasis between eight viral genes. Proc Natl Acad Sci U S A. 117:31519–31526. 33203681 10.1073/pnas.2012331117 PMC7733830

[msad204-B72] Zhao H , Lu L, Peng Z, Chen L-L, Meng X, Zhang C, Ip JD, Chan W-M, Chu AW-H, Chan K-H, et al 2022. SARS-CoV-2 Omicron variant shows less efficient replication and fusion activity when compared with Delta variant in TMPRSS2-expressed cells. Emerg Microbes Infect. 11:277–283. 34951565 10.1080/22221751.2021.2023329 PMC8774049

