# Supplementary material for: Intra- vs. Interhost Evolution of SARS-CoV-2 Driven by Uncorrelated Selection—The Evolution Thwarted
Source: Mol Biol Evol. 2023 Sep 14;40(9):msad204. doi: 10.1093/molbev/msad204 (PMC10521905; doi:10.1093/molbev/msad204)
Supplement: msad204_Supplementary_Data [file msad204_supplementary_data.zip › SI Appendix.pdf]

Supplementary Information for

Intra- vs. inter-host evolution of SARS-CoV-2 driven by uncorrelated selection - The evolution thwarted

Supplementary Figures

T27049C

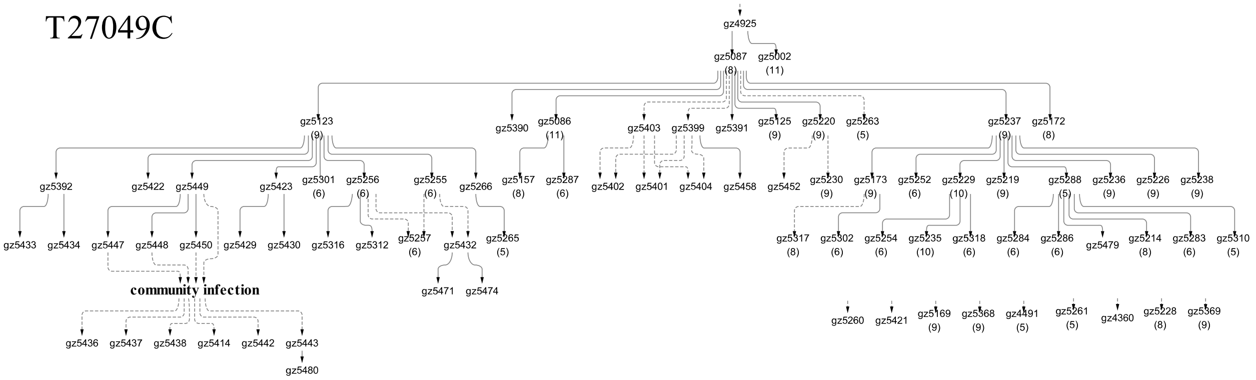

Fig. S1. The continual spread of mutation T27049C. The mutation occurs in more than 41 of all the 79 patients and its frequency (percentage) is shown in the parenthesis. T27049C may be a Type III mutation as it occurs in 41 patients, but at low iSNV frequencies of 5% to 11%. In other words, T27049C has limited within-host proliferation but appears to be good at transmission. The direct donor-recipient transmission is represented by solid arrows (high confidence) or dash lines (with ambiguity).
